# Rice mutants, selected under severe drought stress, show reduced stomatal density and improved water use efficiency under restricted water conditions

**DOI:** 10.3389/fpls.2024.1307653

**Published:** 2024-04-23

**Authors:** Chutima Phunthong, Mutiara K. Pitaloka, Cattleya Chutteang, Siriphat Ruengphayak, Siwaret Arikit, Apichart Vanavichit

**Affiliations:** ^1^ Rice Science Center, Kasetsart University, Nakhon Pathom, Thailand; ^2^ Research Center for Applied Botany, National Research and Innovation Agency, Cibinong, West Java, Indonesia; ^3^ Department of Agronomy, Faculty of Agriculture Kamphangsaen, Kasetsart University, Nakhon Pathom, Thailand

**Keywords:** *Oryza sativa* L., stomatal density and size, stomatal mutants, water use efficiency, water-saving rice, shoot biomass

## Abstract

**Introduction:**

Rice is among the least water-use-efficient crops, and rice plants utilise most of their water uptake for transpirational cooling via stomata. To improve water-use efficiency (WUE) in rice, reducing stomatal density and size could help optimise transpiration and photosynthesis.

**Methodology:**

In this study, we compared two series of purple rice stomata mutants: the Stomatal Model Mutant (SMM) identified by microscopic observation of flag-leaf stomata, and the Drought-selected Model Mutant (DMM) generated through screening under severe water stress. After undergoing two rounds of severe water stress between -60 to -80 Y_m_, right before the R_1–2_ reproductive stage, three DMMs were selected based on their rapid recovery rate and % filled-grain percentage.

**Result:**

The three DMMs displayed 618–697 stomatal units per mm2, similar to the SMMs low-density stomata mutant (JHN 8756 (LD)). Furthermore, the four SMMs, three DMMs and the Jao Hom Nin wild type (JHN WT) were treated with two restricted water condition schemes from seedlings to harvest. The total amount of irrigation and precipitation during the experiment was 78.1 L/plant (69.1 mm/plant) for the less restricted water condition (LR) and 47.5 L/plant (42 mm/plant) for the more restricted water condition (MR). Water condition treatments had no effects on stomatal density and stomatal index. In contrast, genotypes and restricted water condition schemes affected plant height, tillers/plant, % filled grains and shoot dry weight (SDW). The three DMMs and the JHN 8756 (LD), the SMM's low-density stomata mutant, displayed greater resilience towards more restricted water conditions than the SMMs and the JHN wild type. Particularly, DMMs were tolerant to more restricted water condition treatments, showing no SDW penalties. Together, the DMMs and the JHN 8756 (LD) displayed higher WUE under these conditions of more restricted water conditions.

**Conclusion:**

A rigorous screening process to distinguish tolerant mutants with a rapid drought recovery rate from severe water stress could pave the way to isolate more mutants with better stomatal functionality and resilience in preparation for imminent climate changes.

## Introduction

**Figure 1:**
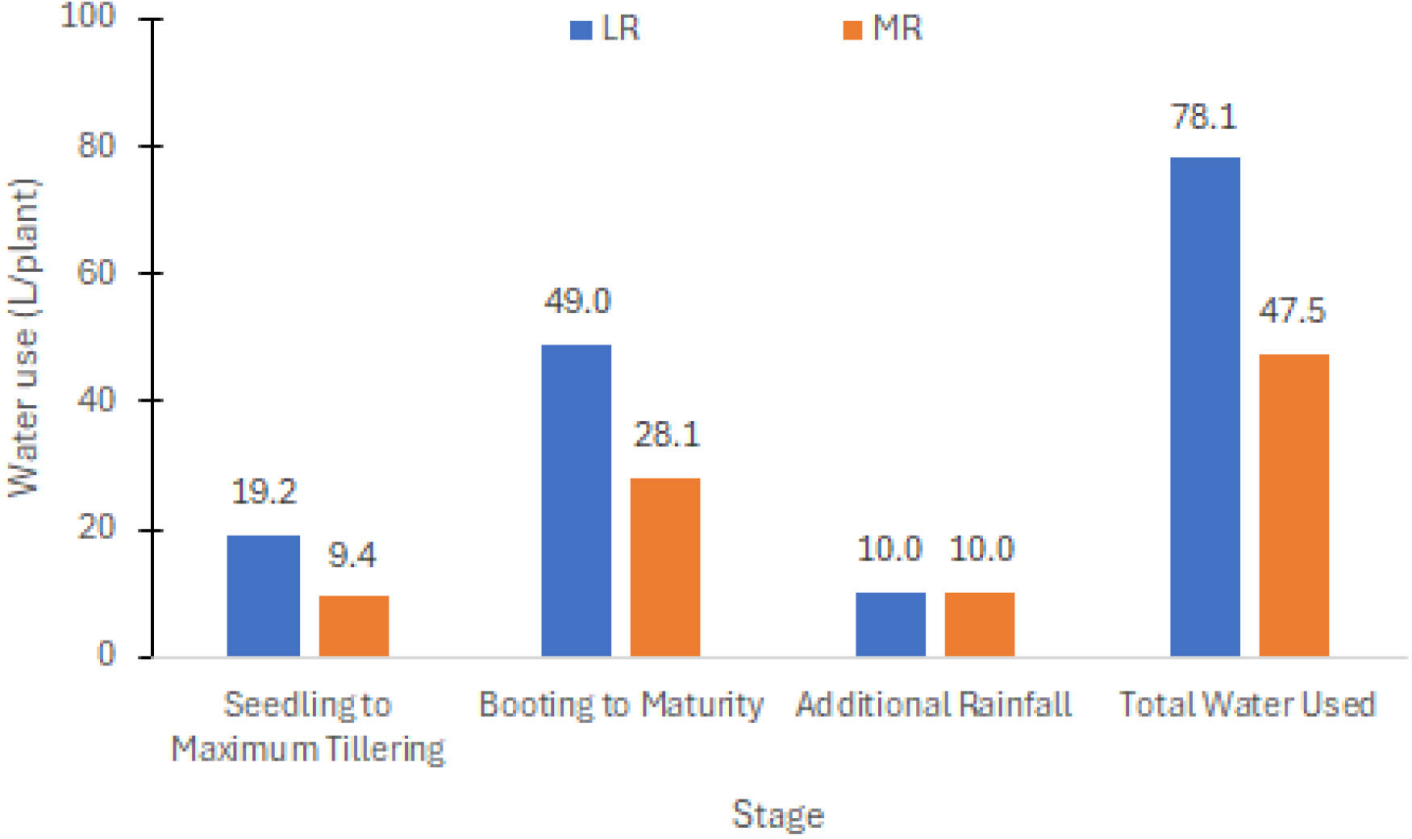
Water usage per plant for the less restricted (LR) and more restricted (MR) irrigation schemes with additional rainfall during the experimental period.

Rice (*Oryza sativa* L.) is a major crop, providing food for billions of people globally (Redfern et al., 2012; Elert, 2014). However, its cultivation is less water-productive, requiring approximately 2,500–5,000 litres of water per kilogram of polished grain produced (Bouman, 2009). According to the Food and Agriculture Organisation, irrigated rice consumes about 34%–43% of the total water used for irrigation globally, partly due to the need to maintain productivity and control weeds (Surendran et al., 2021). Nonetheless, rice loses much of its water through evapotranspiration, comprising both evaporation and transpiration. Transpiration is controlled by several thousand microscopic porous organs called stomata, which are found in all plant leaves and floral parts. These organs are distributed throughout the phyllosphere, including leaf surfaces, leaf sheaths (stems), floral parts and even tiny anthers (Bertolino et al., 2022). Stomatal traits vary between eudicots and monocots. Arabidopsis and green peas displayed kidney-shaped guard cells, while rice and wheat showed dumbbell-shaped guard cells (Bertolino et al., 2019; **Figure 1**). From the micrograph with the same scale bar, we observed rice had the densest and smallest stomata of all plant species. Additionally, rice stomata contain four papillae on guard cells (Pitaloka et al., 2022).

Understanding of how stomatal development is regulated has rapidly been elucidated via discovered genes and molecular physiology affected by altering stomata (Hughes et al., 2017; Raissig et al., 2017; Caine et al., 2018; Schuler et al., 2018). In Arabidopsis and rice, stomatal density is controlled by EPIDERMAL PATTERNING FACTORs (EPF) regulating epidermal cell development (Hashimoto-Sugimoto et al., 2013; Bertolino et al., 2019). Two EPFs, EPF1 and EPF2, both negatively regulate stomatal density (Hara et al., 2007; Hunt and Gray, 2009), while EPFL9 and EPFL10 were positive regulators of stomatal development in rice (Yin et al., 2017).

Stomata function as gatekeepers, regulating the opening and closing of stomatal pores via guard cells in response to light, vapour pressure deficit (VPD), CO_2_ and temperature dynamics to control gaseous exchange. On the one hand, dilation of stomatal aperture enriches plants with CO_2_ for photosynthesis but leads to water loss and cooling. On the other hand, stomatal closure conserves water and reduces transpiration during water stresses but hinders CO_2_ uptake. Furthermore, prolonged heat combined with water stresses may increase canopy temperature and disrupt normal development. These stomatal traits may impact water use efficiency, drought and heat tolerance. Throughout the plant growth cycle, stomatal regulation is resilient to changing light, CO_2_, temperature and VPD conditions (Mustilli et al., 2002; Casson and Gray, 2008; Caine et al., 2018; Pitaloka et al., 2022). Through stomatal functioning, these environmental factors certainly affect crop productivity and water-use efficiency (WUE), determined by the amount of biomass produced per unit volume of water used.

Genetic improvement to enhance WUE is essential in rice, which consumes 34%–43% of irrigation water worldwide (FAO; Surendran et al., 2021). Overexpressing EPF1 markedly reduced stomatal density and improved WUE in barley and rice (Hughes et al., 2017; Caine et al., 2018). In addition, CRISPR/Cas9-mediated knockouts of stomagen and EPFL10 generated rice with 25% and 80% lower stomatal density than the wild type, respectively. However, only moderate stomatal density from EPFL10 lines yielded rice with drought tolerance and optimised photosynthesis (Karavolias et al., 2023). In contrast, a targeted mutation of ASD16, an Indian rice variety, with novel alleles edited by CRISPR/Cas9-OsEPF1, resulted in increasing stomatal density (54%–95%), stomatal conductance (60%–65%), transpiration rate (58%–62%) and photosynthetic rate (14%–31%) compared to the ASD16 wild type (Rathnasamy et al., 2023). Nonetheless, the authors failed to show the relationship between the expressivity of the EPF1 and stomatal density among the mutant progenies. Although some transgenic rice varieties have shown improved drought tolerance and photosynthesis, their deployment in paddy fields has been subjected to high regulation in many rice-growing countries. As an alternative approach, we explored natural genetic variation and stabilised mutant populations for selective breeding.

Insight into natural genetic and physiological variations in stomatal density, WUE and water-stress tolerance has been essential for marker-assisted breeding to optimise stomatal traits and functioning. KDML105-CSS lines exhibited improved plasticity responses to enhance WUE and net photosynthesis by reducing abaxial stomatal density while increasing adaxial stomatal density under drought stress (Lertngim et al., 2023). Among 235 rice accessions, the genome-wide association revealed five QTLs controlling stomata density, showing nine candidate genes, but none was a known EPF gene (Phetluan et al., 2023). Consequently, we decided to pursue forward genetics by isolating stomatal mutants to link stomatal traits with water-use efficiency in rice.

Successful conventional breeding through marker-assisted selection relies heavily on available genetic variation and tightly linked functional markers. However, natural variation in stomatal traits is limited in rice, as it is a self-pollinating crop. Therefore, the narrow genetic variation in stomatal traits has been a critical limiting factor for conventional rice breeding. Mutagenesis induced by radiation or chemicals can rapidly trigger structural and nucleotide changes in the genome, leading to both random and spontaneous mutations that may result in phenotypic gain and be utilised in breeding programs (Ruengphayak et al., 2015; Kamolsukyeunyong et al., 2019). Chemical and physical mutagenesis have been used to induce mutations and create novel resistance and trait modifications in various crops, contributing to the development of new resistant varieties. In rice, induced mutations have been reported using gamma irradiation (Wu et al., 2005; Till et al., 2007), ethyl methanesulfonate (EMS) (Wu et al., 2005), fast neutron (FN) (Wu et al., 2005; Ruengphayak et al., 2015) and ion beam (Yamaguchi et al., 2009). The effects of FN mutagenesis on structural genomic changes have been reported in soybean (Bolon et al., 2011, 2014; Campbell et al., 2016), Arabidopsis (Li et al., 2002) and rice (Ruengphayak et al., 2015; Li et al., 2017). Deletions were also identified as the main structural rearrangements induced by FN mutagenesis (Bolon et al., 2011, 2014; Li et al., 2002). In soybeans, FN treatment induced chromosomal rearrangement near the target gene (Campbell et al., 2016). At the nucleotide level, FN mutagenesis generated single nucleotide polymorphisms (SNPs) in the indica rice cultivar Jao Hom Nin (JHN) (Ruengphayak et al., 2015) and japonica rice cultivar Kitaake (Li et al., 2017). In Kitaake, SNPs were the most abundant mutations, accounting for 48% of the total mutations and 58% of the SNPs were located within rice genes (Li et al., 2017). Structural variation in a coding sequence may create functional mutations; for example, tandem duplication in the waxy gene in rice (Wanchana et al., 2003), structural rearrangement in the NAP1 gene for gnarled trichomes in soybean (Campbell et al., 2016) and haplotype change in OsFRO1 in a rice mutant tolerant to iron (Fe) toxicity (Ruengphayak et al., 2015).

JHN purple rice cultivar was induced using FN to generate a large-scale, stabilised mutant population until the M_10_ generation (FNMP) (Ruengphayak et al., 2015). The FNMP has accumulated unique and spontaneous genetic variations for trait and gene discoveries in a sub-population called the Mutant Core Collection (MCC), comprising 216 M_6_ mutants selected based on extreme phenotypic variation, including altered stomatal size or density. We previously isolated four distinct stomatal traits, including high stomatal density (JHN 2447 (HD), 858 stomata/mm^2^), low stomatal density (JHN 8756 (LD), 651 stomata/mm^2^), large-sized stomata (JHN 826 (LS), 679 stomata/mm^2^) and small-sized stomata (JHN 3117 (SS), 746 stomata/mm^2^). Rice with low-density stomata [JHN 8756 (LD)] and small-sized stomata [JHN 3117 (SS)] demonstrated greater adaptability to drought stress, thereby better maintaining biomass, grain yield and harvest index (Pitaloka et al., 2022). To confirm the linkage between drought tolerance and stomatal density, we further isolated three best-drought recovery mutants from 1,000 randomly chosen FNMP using two rounds of screening under severe water stress conditions.

## Materials and methods

### Selected stomatal mutants from a large mutagenised population

The Jao Hom Nin(*Oryza sativa L.* cv. Jao Hom Nin), referred to Jao Hom Nin wild type (JHN WT), is a photoperiod-insensitive, low-amylose, purple rice cultivar developed by the Rice Science Center, Kasetsart University, Thailand. The large FNMP was generated from 100,000 genetically pure JHN seeds as described (Ruengphayak et al., 2015). We selected seven mutant lines: four based on their stomatal traits and three on extreme water stress conditions. The four Stomatal Model Mutants (SMM) are High Density [JHN2447, JHN 2447 (HD)], Low Density (JHN8756, LD), Small Size (JHN3117, SS), and Large Size (JHN826, LS) – which were microscopically derived from the 216 M_6_ Mutant Core Collection (M_6_ MCC) (Ruengphayak et al., 2015; Pitaloka et al., 2022).

We randomly chose 1,000 M_6_ FNMP (1K) for two rounds of screening under severe water stress conditions. Three plants were grown per line in a plastic pot filled with 4.5 kg of clay. Soil water potential (Ψ_m_) and Volumetric Moisture Content (VMC) at 44 ml per kg of soil were individually controlled between -60 to -80 Ψ_m_, right before the R_1–2_ reproductive stage for the recovery rate and % filled grain. The target VMCs were monitored and maintained for 14 days using a tensiometer placed 12 cm below the soil surface. After returning to normal, we visualised the recovery rate from drought and % filled grain at maturity. We compared with the JHN WT and selected a group of mutants with a better recovery rate and % filled grain from the severe water stress conditions for the next round. After two rounds of selection, only three M_7_ mutants, which exhibited high recovery rates and grain yield, were designated as Drought-selected Model Mutants (DMM) (**Supplementary Table 1**). The SMM and DMM were evaluated for physiological and agronomic traits and WUE (**Supplementary Tables 2**–**4**). All seven mutants generally performed similarly to the JHN WT in flooded-irrigation conditions by showing similar days to flowering (DFT) and harvesting (DTH), having less height but more tiller/plant than JHN WT (**Supplementary Table 3**; **Supplementary Figure 1B**).

### Design of experiments

We designed two restricted water condition regimes to create different water stress conditions, comparing WUE among four SMMs, three DSMs, and JHN WT, the grandparents of these mutants. We investigated stomatal traits and physiological responses to the treatments at the whole-plant level in large cement tanks. The average air temperature ranged between 30°C–40°C during the experimental period from February to July 2023.

**Restricted water condition schemes:** Rice seeds from four SMMs, three DMMs and JHN control were pre-germinated on 200-well plastic trays. Fifteen-day-old seedlings were then transplanted into large concrete tanks in the experimental field at Kasetsart University, Kamphang Saen Campus. The round concrete tank had dimensions of 1.2 m (diameter) × 0.8 m (height) and could hold 750 kg of paddy soil mix. We grew 24 seedlings in each tank with a spacing of 20x20 cm² until harvest. The average air temperature ranged between 35°C and 40°C throughout the experimental period from February to July 2023.

**Irrigation conditions:** We implemented two restricted water condition schemes based on soil moisture levels: a) maintaining volumetric soil moisture content at 40%–50% for less-restricted water conditions (LR) and b) maintaining it at 30%–40% for more-restricted (MR) water conditions throughout the entire crop cycle. Physiological and agronomic traits of SMM, DMM and JHN wild type were monitored under these two restricted water condition schemes until harvest.

We manually added 21–42 and 21–83 litres of water per plant for LR and MR, respectively, to maintain the target soil moisture contents. Rainfall collectors were placed near the experimental site to monitor rainfall during testing. The experiment was designed as a split-plot design in three replications, with the main-plot factors LR and MR serving as the two water condition schemes. Seven selected mutants and the JHN control were treated as the sub-plot factors arranged in a Completely Randomised Block (CRB). Each soil tank was considered an experimental unit, resulting in 48 experimental units comprising two water condition schemes, eight varieties and three replications.

**Monitoring soil moisture and temperature:** Soil moisture and temperature were daily monitored at a depth of 12 cm below the soil surface starting at 3:00 p.m. using a soil moisture meter (Field Scout TDR150). The main plot treatments for LR and MR schemes were manually irrigated, aiming to maintain volumetric soil moisture content (VMC) at 30%–40% and 40%–50%, respectively. These water levels were deemed sufficient for sustaining plant growth from the vegetative to the reproductive stages. Seasonal rainfall recorded at the experimental site amounted to approximately 214 mm (equivalent to 10 ml per plant), contributing to the overall water usage. The average water consumption per plant in LR and MR conditions was 69.1 mm and 42.0 mm, or 78.1 L/plant and 47.5 L/plant, respectively (see **Figure 1**). Throughout the reproductive to harvest stages, average soil temperatures ranged between 33°C–37°C for both water conditions.

### Data collection

We collected physiological and agronomic traits for flag leaves at R_1–2_ and pre-harvest stages from eight plants per treatment for subsequent statistical analysis.

#### Reproductive stage

a) **Leaf rolling score** was visually assessed between 11:00 and 12:00, following the method outlined by O’Toole and Cruz (1979).
b) **Agronomic traits:**
**-** Days to flowering Time (FD): This metric represents the duration from germination to the point when 50% of the panicles have fully bloomed.
**-** Delayed flowering (Delay_FD): The differences in FDs for the same rice variety under less restricted (LR) and more restricted (MR) water conditions was computed as follows:
$○\%\text{Delay}\_\text{FD}=\frac{\text{FD}\left(\text{LR}\right)-\text{FD}\left(\text{MR}\right)}{\text{FD}\left(\text{LR}\right)}\text{x}100$

**-** Plant height (PH): This parameter measures the distance from the soil surface to the top of the canopy.
**-** Decreased plant height: The differences in PH for the same rice variety under less restricted (LR) and more restricted (MR) water conditions were calculated as follows: The percentage of reduce_PH was calculated as follows;
$○\%\text{Reduce}\_\text{PH}\;=\frac{\text{PH}\left(\text{LR}\right)-\text{PH}\left(\text{HR}\right)}{\text{PH}\left(\text{LR}\right)}\text{x}100$

c) **Chlorophyll fluorescence (Cf):** Chlorophyll fluorescence (cf) was monitored regularly between 21:30 and 22:00 to avoid light interference and when the air temperature was more stable. Measurements were carried out during the dark period to determine minimal (Fo), maximal (Fm), and maximal variable fluorescence (Fv = Fm − Fo) of the leaf under steady-state conditions with a leaf-clip holder (2030B, Heinz Walz). The dark-adapted leaves were irradiated by a weak modulated measuring beam set at 2.0 µmol photons m-2 s-1 to determine Fo and a saturating flash set at 3,000 mmol photon m-2 s-1, 20 kHz within 0.8 s, to define Fm, following Maxwell et al. (2000).

$$\text{Maximum quantum efficiency}=\left({\text{F}}_{\text{m}}-{\text{F}}_{0}\right)/\text{Fm}={\text{F}}_{\text{v}}/{\text{F}}_{\text{m}}$$

*-* F_0_: the minimum quasi-dark fluorescence yield,
*-* F_m_: the maximum total fluorescence yield,
*-* F_v_: the variable fluorescence = F_m_-F_0_
d) **SPAD index:** The SPAD index reflects the average greenness obtained from five positions on the flag leaf using a portable chlorophyll meter (SPAD-502, Minolta Camera, Japan). Responses were compared between two light spectra: the 650 nm maximum-chlorophyll absorbable spectrum (Red LED) and the 940 nm (Infrared LED) used as a reference. We measured the SPAD index 1, 7, 14 and 21 days after the R_2_ stage.
e) **Specific Leaf Weight (SLW):** SLW measures the thickness of flag leaves during the R_1–2_ stage. The total area (LA) of six flag leaves was recorded using the Image Gal Application, after which they were dried completely to obtain their dried weight (LW).

$$\begin{array}{l} SLW=LW/LA\\ \;LA=\text{Total leaf area}\\ \;LW=\text{Total leaf-dried weight} \end{array}$$

f) **Microscopic observation of stomatal traits:** Fully expanded flag leaves were collected from three plants (biological samples) of each line. Three leaf segments from the adaxial and abaxial leaf surfaces were imprinted on glass slides using dental resin (Coltene Whaledent, Switzerland) and left to set before removing the leaf and applying clear nail varnish to the resin. Microscopic epidermal images were captured from the middle area of leaves using camera-mounted light microscopes (Leica, DM750-ICC50 HD and 13613242/0.17 PLAN 40X/0.65) at 40X magnification (2,048 × 1,536 pixels or 0,529 x 0,264 mm.) per leaf (**Supplementary Figure 1A**). Cell counting was conducted within six fields of view (FOV) as the estimate was more stabilised (**Supplementary Table 9**). Using ImageJ software (Fiji v. 1.51u), stomatal cells within each field of view were counted and converted into the total stomatal density per mm^2^ (Pitaloka et al., 2022). The Stomatal Index (*si*) was calculated as follows:

Stomatal Index (%)=(S/S+E)×100

S = The number of stomata per unit area.

E = The number of epidermal cells in a unit area.

#### Traits collected during pre-mature stage

a) % Filled Grain (FG) represents the percentage of filled grains over the total filled and unfilled grains, averaged over 18 panicles from each line. The percentage of reduce_FG was calculated as follows;

$$\%\text{Reduce}\_\text{FG }=\;\frac{\text{FG}\left(\text{LR}\right)-\text{FG}\left(\text{HR}\right)}{\text{FG}\left(\text{LR}\right)}\text{x}100$$

b) Shoot Dry Weight (SDW) is the biomass (g) above ground collected at maturity. The percentage of reduce_SDW was calculated as follows;

$$\%\text{ReduceSDW}\;=\;\frac{\text{SDW}\left(\text{LR}\right)-\text{SDW}\left(\text{HR}\right)}{\text{SDW}\left(\text{LR}\right)}\text{x}100$$

c) The number of tillers per plant (Till) is averaged over six plants per plot. The percentage of reduced tiller per plant was calculated as:

$$\%\text{Reduced Till}=\frac{\text{Till}\left(\text{LR}\right)-\text{Till}\left(\text{HR}\right)}{\text{Till}\left(\text{LR}\right)}\text{x}100$$

d) Water Use Efficiency (WUE) is the dried weight (14%) of biomass (g) per total litre of water used per plant (g/L). WUE was calculated as mg/100 ml. The percentage of induce_WUE was computed as follows;

$$\%\text{Induce}\_\text{WUE}=\frac{\text{WUE}\left(\text{HR}\right)-\text{WUE}\left(\text{LR}\right)}{\text{WUE}\left(\text{LR}\right)}\text{x}100$$

e) Plant height (PH) is the distance (cm) from the ground level to the canopy top, averaged over six plants per plot. The percentage of decrease_PH was calculated as follows:

$$\%\text{Reduce}\_\text{PH}=\frac{\text{PH}\left(\text{LR}\right)-\text{PH}\left(\text{MR}\right)}{\text{PH}\left(\text{LR}\right)}\text{x}100$$

### Statistical analysis

Statistical analysis was performed using analysis of variance (ANOVA) in R version 3.4.3 to determine the main and interaction effects. Treatment means were compared using the least significant difference (LSD) to assess whether they were significantly different at the 0.05 probability level. Correlation analysis was conducted, calculating a two-tailed Pearson correlation coefficient with significance levels of 0.05 and 0.01.

## Results

### Isolating drought-tolerant mutants

**Figure 2:**
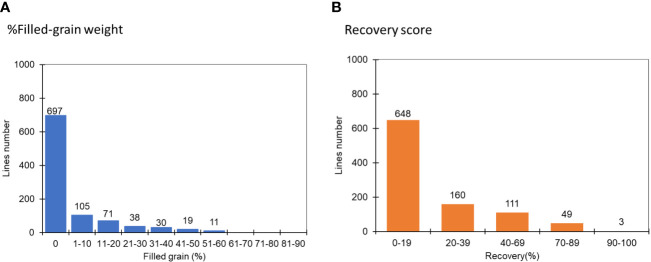
The distribution of recovery rate and filled-grain weight at the R_1-2_ stage among 1000 FNMP in response to extreme water stress conditions in the first round of screening: **(A)** % Filled-grain weight, **(B)** Recovery score, where 1 is equivalent to 90-100% and 9, 0-19%.

We randomly selected 1,000 lines from FNMP grown under severe water stress conditions to identify drought-tolerant mutants. In the first selection round, which focussed on recovery rate and grain yield, it was observed that over 60% of FNMP lines exhibited a recovery rate of less than 19% without seed set (**Figure 2**). In contrast, JHN WT showed a recovery of nearly 40% of its full-grain weight. Only 30 mutants that performed better than JHN WT were selected and advanced to the second round of screening under severe water stress conditions.

**Figure 3:**
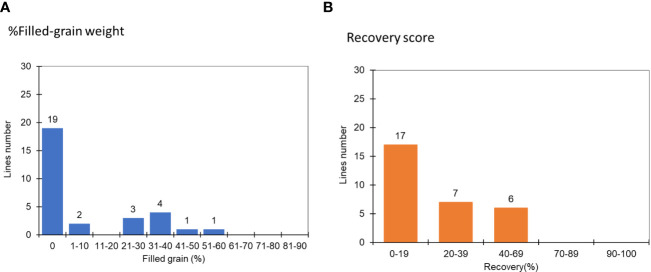
The distribution of recovery rate and filled-grain weight at the R1-2 stage among 30 FNMP in response to extreme water stress conditions in the second round of screening: **(A)** % Filled-grain weight, **(B)** Recovery score, where 1 is equivalent to 90-100% and 9, 0-19%.

**Figure 4:**
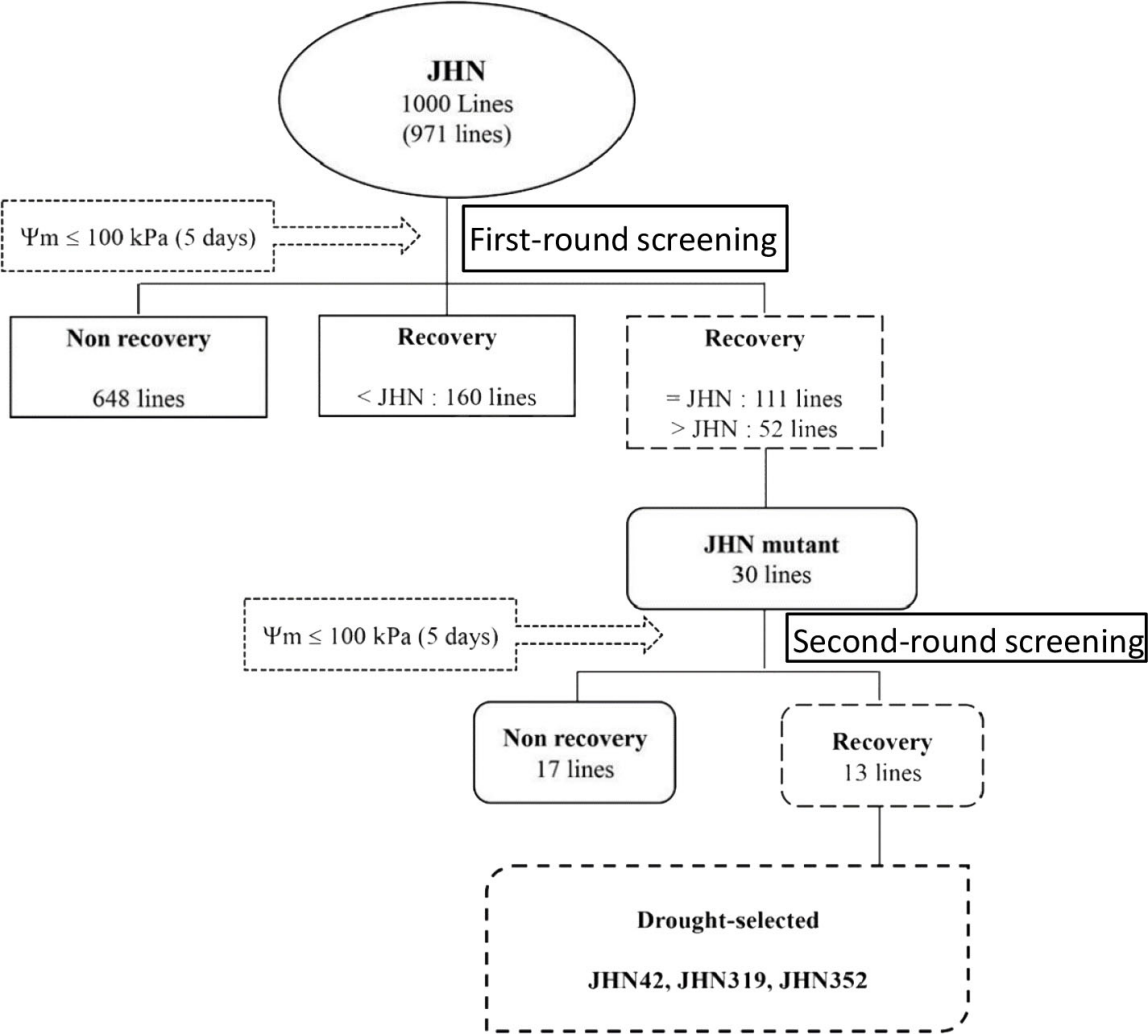
Schematic diagram illustrating two rounds of selective screening under extreme water stress conditions in 1,000 FNMP (first round) and 30 lines (second round).

In the second cycle, seeds from the previously selected mutants were sown under similar severe water stress conditions. The results indicated that 63% of the chosen lines did not set seed, and 53% exhibited a recovery rate of less than 20% (**Figure 3**). From the pool of 30 lines, the top three mutants – JHN 42, JHN 319 and JHN 352 – outperformed JHN WT. These mutants were selected for further comparison with SMM in the field evaluation for water use efficiency (WUE) (**Figure 4**; **Supplementary Table 1**).

### Modification of stomatal density

**Figure 5:**
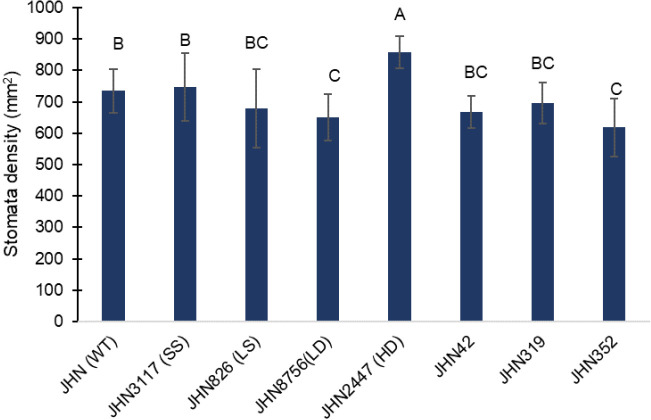
Stomatal density among stomatal mutants and JHN WT, averaged over two water conditions, LR (Less restricted water condition) and MR (More restricted water condition). Genotypic means are shown with standard deviations (±SD) and labelled with alphabetical combinations. For each water condition, the genotypic means shared similar alphabets were statistically indifferent (ns). JHN WT (wild type) and four stomatal model mutants (SMM) are JHN826 (large-sized stomata), JHN3117 (small-sized stomata), JHN8756 (low-density stomata), JHN2447 (high-density stomata), and three drought-selected stomatal model mutants (DMM), JHN42, JHN352, JHN319.

Water conditions generally did not affect stomatal density (*sd*), stomatal index (*si*) or specific leaf weight (*slw*). However, more restricted water conditions partially reduced the *sd* or *si* of all mutants, including JHN WT, but they were insignificant (**Figure 5**; **Supplementary Table 2**). Nonetheless, the *sd* and *si* among mutant lines differed significantly and independently from the water conditions. Among the SMMs, JHN 2447 (HD) showed the highest *sd* at 858±52 stomata per mm^2^ (**Figure 5**; **Supplementary Table 2**). In contrast, JHN 8756 (LD) showed the lowest *sd* at 651±74 stomata per mm^2^. Among the DMMs, *sd* exhibited a narrow range between 618±92 and 697±65 stomata per mm^2^ and between 29±2 and 35±5 for *si*. Additionally, the stomatal sizes (*sz*) of these mutants were classified into small (JHN 3117, SS), large (JHN 826, LS), and intermediate for the rest (**Supplementary Table 2**). From these results, all DMM lines were similar to JHN 8756 with low *sd*, *si*, and *sz*. We further investigated the biomass accumulation and WUE of SMMs and DMMs.

### Biomass accumulation and WUE

**Figure 6:**
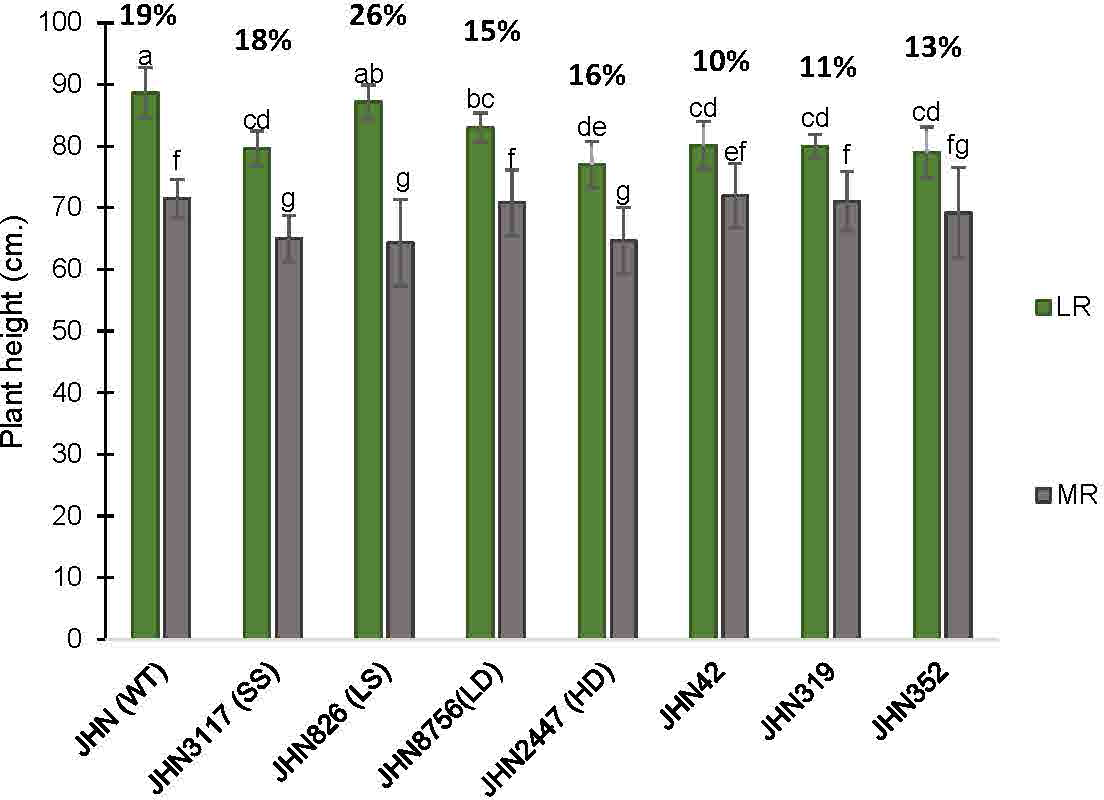
The percentage plant height (PH) reduction collected from JHN WT and seven stomatal mutants were grown under less restricted (LR) and more restricted water conditions (MR). Genotypic means are shown with standard deviations (±SD) and labelled with alphabetical combinations. For each water condition, the genotypic means shared similar alphabets were statistically indifferent (ns). JHN WT (wild type) and four stomatal model mutants (SMM) are JHN826 (large-sized stomata), JHN3117 (small-sized stomata), JHN8756 (low-density stomata), JHN2447 (high-density stomata), and three drought-selected stomatal model mutants (DMM), JHN42, JHN352, JHN319. $\%\text{Reduce}\_\text{PH}=\frac{\text{PH}\left(\text{LR}\right)-\text{PH}\left(\text{HR}\right)}{\text{PH}\left(\text{LR}\right)}\text{x}100$ .

**Figure 7:**
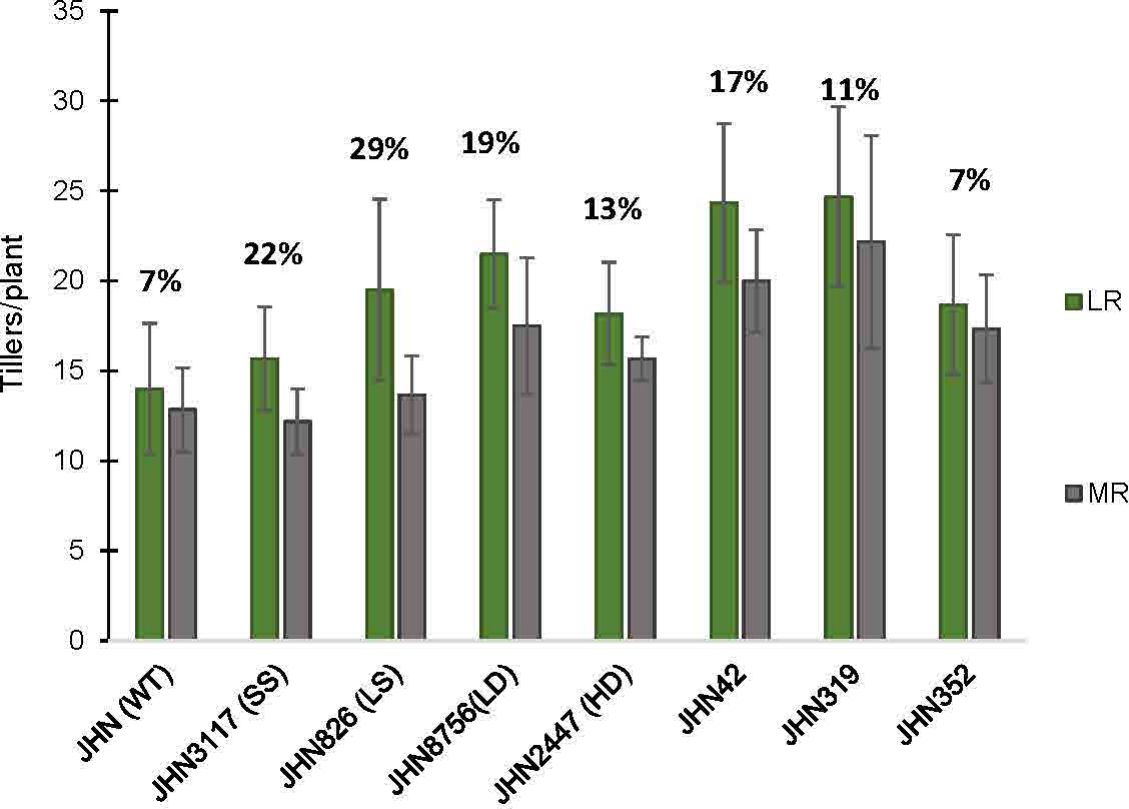
The percentage tillers per plant reduction (Till) collected from JHN WT and seven stomatal mutants were grown under less restricted (LR) and more restricted water conditions (MR). Genotypic means are shown with standard deviations (+SD) and labelled with alphabetical combinations. For each water condition, the genotypic means that shared similar alphabets were statistically indifferent (ns). JHN WT (wild type) and four stomatal model mutants (SMM) are JHN826 (large-sized stomata), JHN3117 (small-sized stomata), JHN8756 (low-density stomata), JHN2447 (high-density stomata), and three drought-selected stomatal model mutants (DMM), JHN42, JHN352, JHN319. $\%\text{Reduce}\_\text{Till}\;=\;\frac{\text{Till}\left(\text{LR}\right)-\text{Till}\left(\text{HR}\right)}{\text{Till}\left(\text{LR}\right)}\text{x}100$ .

In this study, treatments with restricted water conditions did not consistently impact the maximum quantum yield of photosystem II and SPAD values (**Supplementary Tables 7**, **8**). Notably, SPAD values were higher in less restricted (LR) than more restricted (MR) water conditions on the first day after booting (DAB) but lower 21 days after. In contrast to maximum quantum yield and SPAD, the increase in greenness in MR on the 21 DAB was more evident among the DMMs, reflecting greater adaptability to water stress (**Supplementary Tables 7**, **8**). Moreover, MR reduced plant height, tillers/plant, % filled grain and delayed flowering (**Figures 6**, **7**; **Supplementary Tables 3**, **4**). Specifically, delayed flowering, plant height reduction and reduced tillers/plant in MR were more severe in JHN 826 (LS), JHN 2447 (HD) and JHN 3117 (SS) than in JHN 8756 (LD) and DMM (**Figure 7**; **Supplementary Table 6**). JHN 826 (LS) mainly drastically reduced plant height and tillers/plant, while JHN 8756 (LD) exhibited a lesser effect.

**Figure 8:**
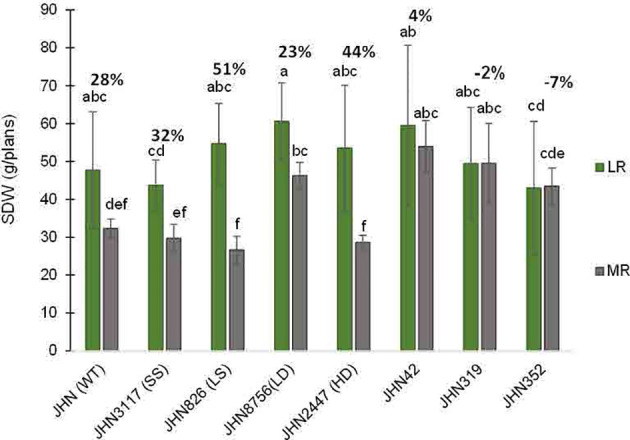
The percentage shoot dry weight reduction (SDW) collected from JHN WT and seven stomatal mutants were grown under less restricted (LR) and more restricted water conditions (MR). Genotypic means are shown with standard deviations (+SD) and labelled with alphabetical combinations. For each water condition, the genotypic means that shared similar alphabets were statistically indifferent (ns). JHN WT (wild type) and four stomatal model mutants (SMM) are JHN826 (large-sized stomata), JHN3117 (small-sized stomata), JHN8756 (low-density stomata), JHN2447 (high-density stomata), and three drought-selected stomatal model mutants (DMM), JHN42, JHN352, JHN319. $\%\text{Reduce}\_\text{SDW }=\;\frac{\text{SDW}\left(\text{LR}\right)-\text{SDW}\left(\text{HR}\right)}{\text{SDW}\left(\text{LR}\right)}\text{x}100$ .

Similarly, DMMs reduced plant height and tillers/plant less in more restricted water conditions (MR). Therefore, JHN 8756 (LD) and DMMs responded similarly to more restricted water conditions. Since tillers/plant and plant height affected shoot dry weight (SDW), water conditions that influenced tillering capacity also impacted shoot biomass, leading to a substantial reduction in SDW (**Figure 8**; **Supplementary Table 5**). In addition, both restricted water conditions and genotypes significantly affected biomass, as evidenced by the notable reduction in SDW (**Figure 8**). Nonetheless, more restricted water conditions affected plant height and tillering capacity, less than above-ground biomass (**Figures 6**–**8**). As DMMs and JHN 8756 (LD) retained higher SDW than others, stomatal density might have played essential roles in conferring resilience against restricted water conditions and water stress.

**Figure 9:**
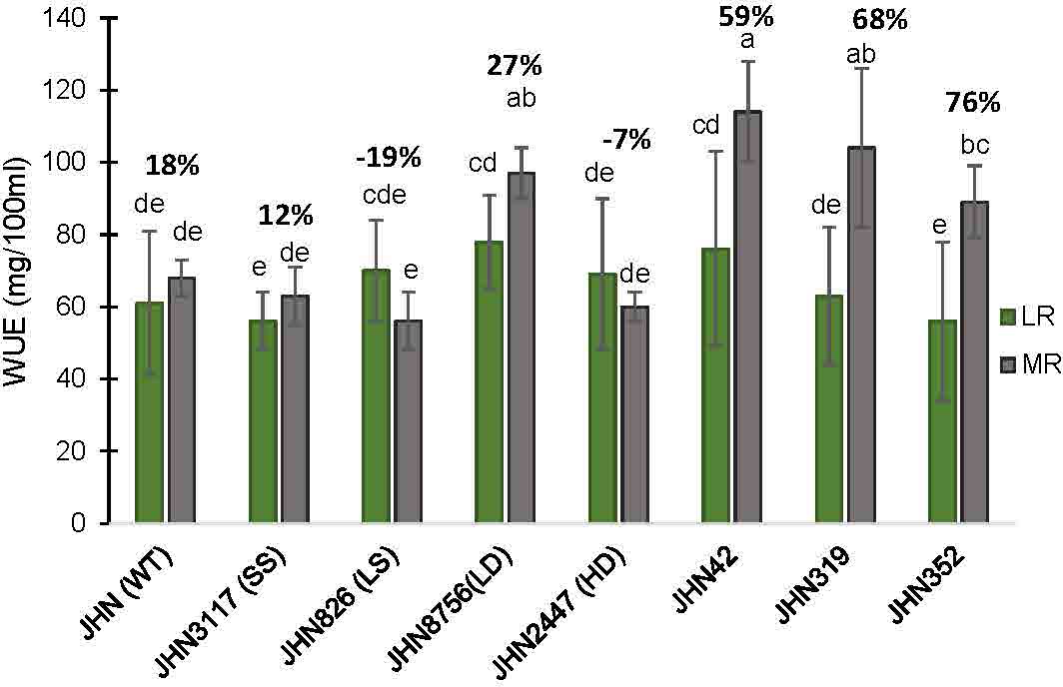
The percentages of increase water use efficiency (WUE) collected from JHN WT and seven stomatal mutants were grown under less restricted (LR) and more restricted water conditions (MR). Genotypic means are shown with standard deviations (±SD) and labelled with alphabetical combinations. For each water condition, the genotypic means shared similar alphabets were statistically indifferent (ns). JHN WT (wild type) and four stomatal model mutants (SMM) are JHN826 (large-sized stomata), JHN3117 (small-sized stomata), JHN8756 (low-density stomata), JHN2447 (high-density stomata), and three drought-selected stomatal model mutants (DMM), JHN42, JHN352, JHN319. $\%\text{Induce}\_\text{WUE}=\frac{\text{WUE}\left(\text{HR}\right)-\text{WUE}\left(\text{LR}\right)}{\text{WUE}\left(\text{LR}\right)}\text{x}100$ .

Restricted water condition treatments and genotypes significantly affected WUE and SDW. DMMs and JHN 8756 (LD) consistently exhibited higher WUE and above-ground biomass (SDW) than JHN 2447 (HD), JHN 826 (LS) and JHN 3117 (SS) under more restricted water conditions (MR) (**Figures 8**, **9**; **Supplementary Table 5**). Among SMMs, JHN 8756 (LD) retained the most negligible penalty in SDW, while DMMs were notably performed even better. Therefore, gaining higher SDW and WUE in more restricted (MR) water conditions may be highly associated with low to intermediate-density stomata.

**Figure 10:**
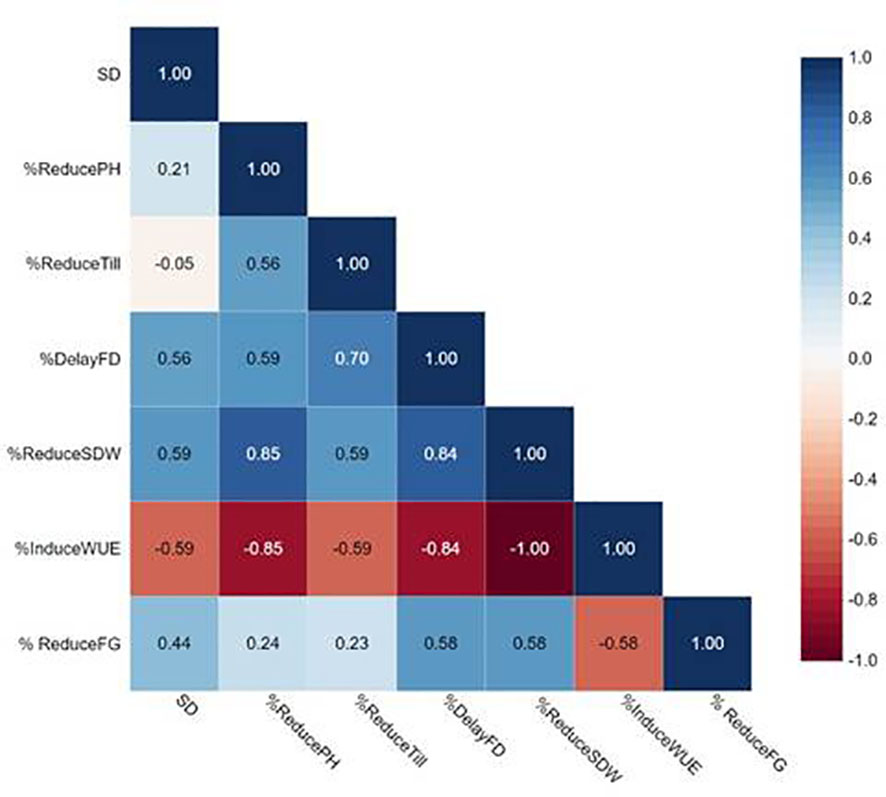
Pearson correlation coefficients among stomatal density (SD), %Reduce_plant height (ReducePH), %Reduce_tillers/plant (ReduceTill), %Reduce_filled grains (FG) (ReduceFG), %Reduce_shoot dry weight (ReduceSDW), and %Induce_water use efficiency (InduceWUE) from stomatal mutants grown under less restricted (LR) and more restricted (MR) water conditions.

**Figure 11:**
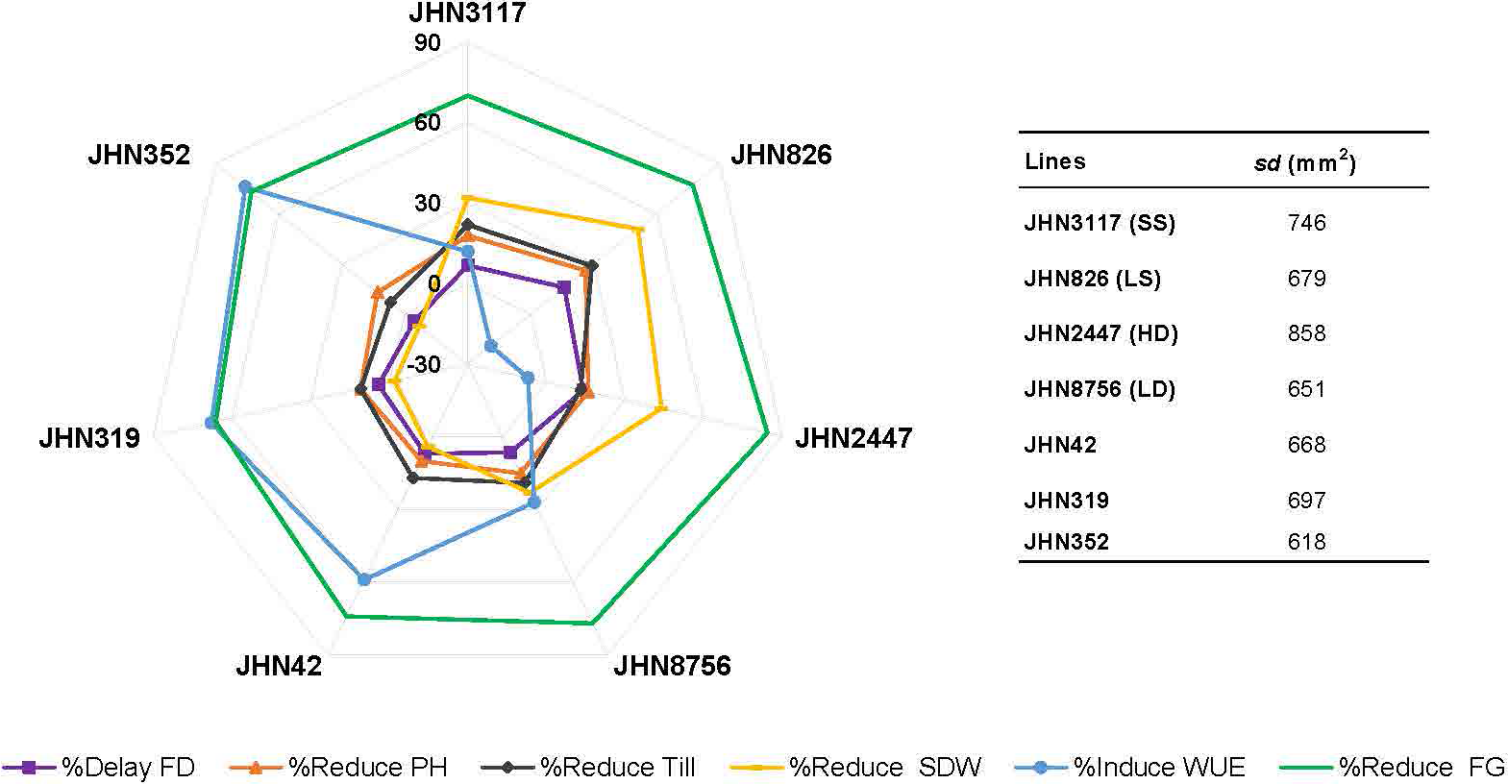
Comparison among seven stomatal mutants for %Reduce_plant height, %Reduce_tillers/plant, %Reduce_filled grains (FG), %Reduce_shoot dry weight, and %Induce_water use efficiency were performed under less restricted (LR) and more restricted (MR) water conditions.

We performed correlation analysis among stomatal mutants for stomatal density (SD), % reduced plant height (%Reduce_PH), % reduced tiller/plant (%Reduce_Till),% delayed flowering (%Delay_FD), % reduced shoot dry weight (%Reduce_SDW), % induced water use efficiency (%Induce_WUE), and % reduced filled grains (%Reduce_FG) (**Figure 10**; **Supplementary Table 6**). SD showed no correlation with any traits in this study, particularly %Reduce_SDW and %Induce_WUE. The fact that DMMs had intermediate to low stomatal density, stomatal density per se did not play vital roles in improving shoot biomass and WUE. Reduced filled grain (%Reduce_FG) showed a strong correlation with delayed flowering (Delayed FD) and reduced shoot dry weight (%Reduce_SDW) but not induced WUE (%Induce_WUE). These results exhibited more water stress conditions created by restricted irrigation improved WUE by reducing delayed flowering and maintaining plant height, tillering and improving shoot dry weight among low-density stomata mutants JHN8756 (LD), and the three DMMs, including JHN 42, JHN 319, and JHN352 (**Figure 11**). Therefore, shoot dry weight, and WUE were strongly influenced similarly by the interaction between stomatal traits, especially stomatal functioning, and water stress conditions.

## Discussion

### Efficient approaches to identify high water use efficient rice

In a previous study, we demonstrated a promising approach to isolate four stomatal model mutants (SMM) from a large-stabilised FNMP based on stomatal densities and sizes using microscopic observations (Pitaloka et al., 2021). The results showed that JHN 8756 (LD) had the highest WUE under restricted water conditions. In this study, we successfully isolated three mutants with strong drought recovery and low-density stomata from a larger random subpopulation of the same FNMP through two successive rounds of selection under severe water stress conditions during the reproductive stage. Which strategies led to more effectiveness in isolating functional mutants with high WUE and water-saving rice? Both approaches relied on induced genetic variation in the 25K M_4_ FNMP (Ruengphayak et al., 2015). The microscopic process relied on existing induced genetic variation in the selected M6 hypervariable subpopulation, comprising 216 mutants accumulating multiple mutations for resistance to biotic and abiotic stresses, grain qualities and morphological alterations through forward and reverse screenings. Such germplasm is a valuable genetic reservoir providing excellent opportunities to seek rare mutants, such as stomatal traits, rather than working on the original FNMP, which is much larger.

Alternatively, the stringent selection for better drought recovery with lower *sd* and higher WUE under severe water stress seems robust (Kumar et al., 2021; Caine et al., 2023; Robertson et al., 2023). Here, we demonstrated that severe water stress conditions during the reproductive stage enabled a general breeder to isolate functional mutants with lower stomatal density without microscopic observation. From the 1K M_6_ subpopulation of FNMP, three elite mutants with high drought recovery were successfully identified through two rounds of screening. These drought-selected mutants (DMM) exhibited low-density *sd* similar to JHN 8756 (LD), which was isolated by selection after microscopic observation.

### Can we breed for more water-saving rice?

Rice production accounts for 34%–43% of irrigation water consumption worldwide, and farmers are expected to face water scarcity due to imminent climate change (FAO; Surendran et al., 2021). Generally, water usage in lowland irrigated rice was reported to be 0.4 g/litre (Bouman et al., 2006). Therefore, cultivating water-saving varieties and adopting water-efficient practices are crucial for ensuring future rice production. The Alternate Wet and Drying (AWD) technique, developed by the International Rice Research Institute (IRRI), has undergone extensive evaluation in Asian countries and has proven highly beneficial for improving water-use efficiency (23%–37%). Additionally, it contributes to reducing greenhouse gas emissions (45%–90%), saving fertiliser, minimising diseases and pests (92%–100%) and enhancing overall water productivity (Bouman and Tuong, 2001; Bouman and Lampayan, 2009; Das et al., 2012; Ishfaq et al., 2020; Mallareddy et al., 2023). The water usage in flooded rice ranged between 1324 and 2250 mm, whereas Alternate Wetting and Drying (AWD) consumed between 1000 and 1151 mm or 20% and 40% less than flooded irrigation practices (Kumar and Rajitha, 2019). In this study, the water usage of the less and more restricted irrigations (LR vs MR) were 1662 and 1011 mm, respectively, equivalent to regular flooded and AWD irrigations.

However, these water-saving cultivation practices have not necessarily improved water-use efficiency in any rice varieties. From the same amount of irrigation, rice with low stomatal density, exemplified by JHN 8756 (LD), produced 16% and 35% more biomass than JHN WT, a popular local rice cultivar, and JHN 2447, a high-density stomatal mutant, respectively, particularly under water stress conditions (Pitaloka et al., 2022). In the current study, the three drought-selected mutants and JHN 8756 (LD) exhibited a 50%–88% and 44% increase in biomass yield compared to JHN WT under the more restricted water condition regime. Additionally, compared to more restricted and less restricted water conditions, these intermediate-to-low-density stomatal mutants exhibited less penalty on biomass yields and higher WUE than JHN WT and other stomatal mutants (**Figure 8**). Therefore, our results strongly support that low-density stomatal rice can even improve water productivity in AWD. Furthermore, evaluating these selected WUE mutants in paddy fields is crucial to determining their grain yield advantage and water productivity under the AWD technique.

### Are water saver mutants less productive under regular irrigation?

All stomatal density mutants performed similarly to the wild-type JHN under less restricted water condition schemes. In a previous study, the four stomatal model mutants (SMMs) were compared between sufficient (37.8 L/plant) and restricted (14.5 L/plant) water conditions (Pitaloka et al., 2022). Under sufficient watering conditions, all SMMs exhibited similar photosynthetic assimilation (*A*), stomatal conductance (*gs*), chlorophyll fluorescence and responsiveness to elevated CO_2_. In addition, the SMMs were also similar in several agronomic traits, including days to flowering, leaf length, leaf width, tillers/plant, plant height and WUE under well-water conditions. In this experiment, we compared the SMMs and three DMMs under less restricted water conditions (LR) (76 L/plant) and more restricted water conditions (47.5 L/plant). Based on similar morphological characteristics and agronomic responses in less restricted water conditions, we can postulate that SMMs and DMMs exhibited normal photosynthetic assimilation and stomatal conductance but differed in transpiration rate based on their differences in stomatal densities. Biomass and WUE of all stomatal mutants were statistically indifferent under less restricted water conditions. In contrast, all low-density stomata mutants, including JHN 8756 (LD) and DMMs, accumulated more biomass and showed higher WUE than the wild type and other SMMs, particularly under more restricted water condition schemes. Thus, we can confirm that under sufficient water conditions, these stomatal mutants (SMMs and DMMs) performed as well as the wild-type JHN, the original variety of the large stabilised mutagenised population.

## Conclusions

We successfully isolated three purple rice mutants adaptable to severe water stress conditions during the reproductive period. These mutants exhibited low stomatal density and achieved higher water-use efficiency, particularly under more restricted water conditions. We demonstrated that rice with lower *sd* was more resilient under water stress conditions by stabilising plant height, tillering capacity, filled grains, retaining higher biomass, and improving water use efficiency.

## Data availability statement

The original contributions presented in the study are included in the article/**Supplementary Material**. Further inquiries can be directed to the corresponding author.

## Author contributions

CP: Data curation, Investigation, Writing – original draft. MP: Investigation, Writing – review & editing. CC: Methodology, Writing – review & editing. SR: Investigation, Project administration, Validation, Writing – review & editing. SA: Resources, Validation, Writing – review & editing. AV: Conceptualization, Funding acquisition, Writing – original draft.
