# Supplementary figures and images for: Rice mutants, selected under severe drought stress, show reduced stomatal density and improved water use efficiency under restricted water conditions
Source: Front Plant Sci. 2024 Apr 23;15:1307653. doi: 10.3389/fpls.2024.1307653 (PMC11075636; doi:10.3389/fpls.2024.1307653)

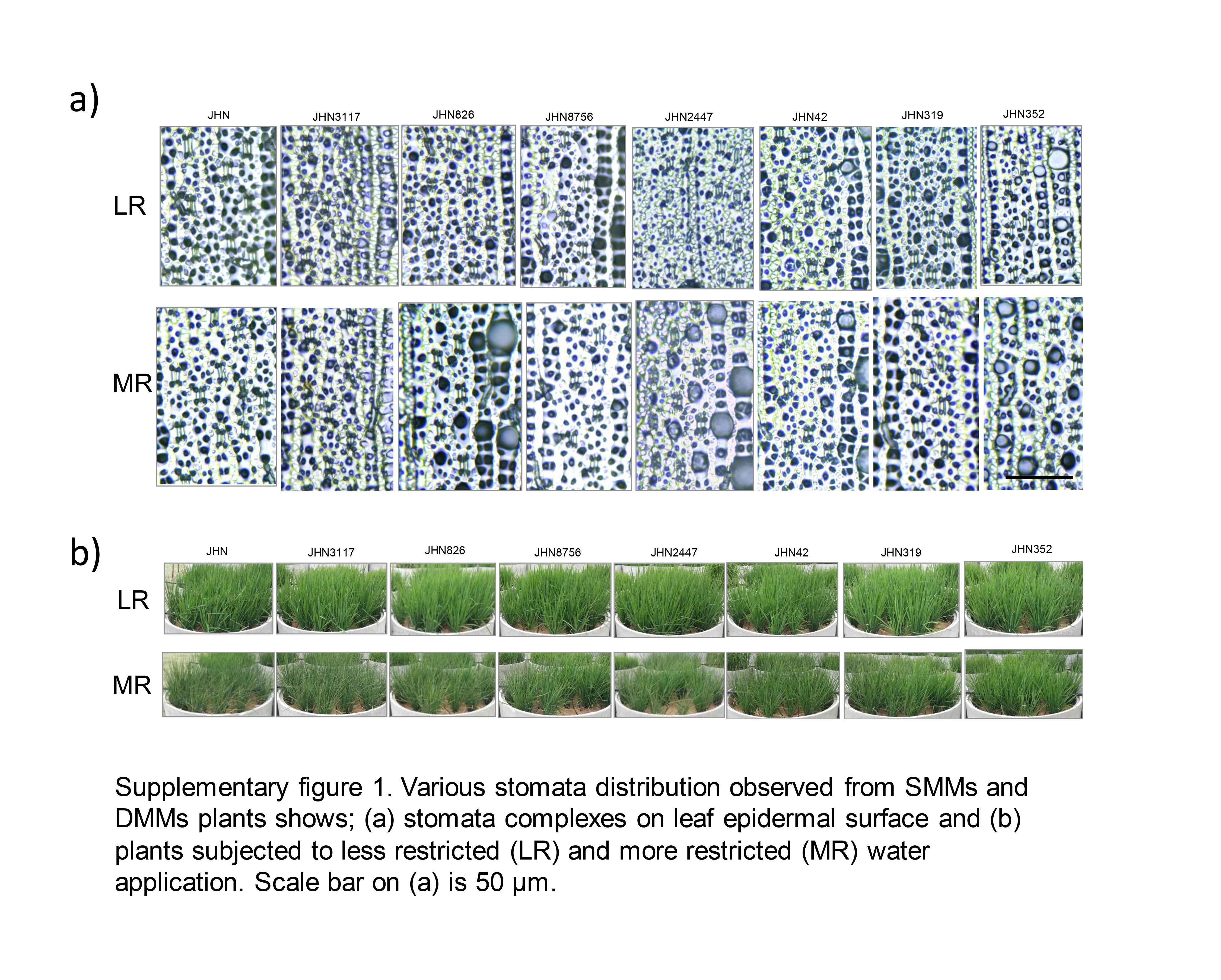

Supplement: Supplementary file 1 [file Image_1.jpeg]
