# Supplementary material for: Rice mutants, selected under severe drought stress, show reduced stomatal density and improved water use efficiency under restricted water conditions
Source: Front Plant Sci. 2024 Apr 23;15:1307653. doi: 10.3389/fpls.2024.1307653 (PMC11075636; doi:10.3389/fpls.2024.1307653)
Supplement: Supplementary file 2 [file DataSheet_1.pdf]

Supplementary Table 1: Recovery scores and % Filled grains among 30 mutants selected under severe water stress conditions.

Lines	Recovery score	std	% Filled Grain	std
JHN42	5	0	31.8	2.6
JHN319	5	0	39.4	1.1
JHN352	5	0	50.8	1.4
JHN3201	5	0	42.6	4.4
JHN40	5	0	27.7	4.6
JHN9002	5	0	35.6	0.2
JHN5547	7	0	31.2	3.7
JHN171	7	0	21.2	0.4
JHN176	7	0	1.1	2.0
JHN2544	7	0	0.0	0.0
JHN3059	7	0	0.0	0.0
JHN314	7	0	1.2	2.1
JHN901	7	0	21.4	1.4
JHN5727	9	0	0.0	0.0
JHN2537	9	0	0.0	0.0
JHN1840	9	0	0.0	0.0
JHN1887	9	0	0.0	0.0
JHN19	9	0	0.0	0.0
JHN221	9	0	0.0	0.0
JHN224	9	0	0.0	0.0
JHN30	9	0	0.0	0.0
JHN3079	9	0	0.0	0.0
JHN3080	9	0	0.0	0.0
JHN3096	9	0	0.0	0.0
JHN3202	9	0	0.0	0.0
JHN339	9	0	0.0	0.0
JHN409	9	0	0.0	0.0
JHN476	9	0	0.0	0.0
JHN75	9	0	0.0	0.0
JHN914	9	0	0.0	0.0

Supplementary Table 2: Stomatal density (*sd*, mm2), stomatal size (*sz,* mm), % stomatal index (*si*) and specific leaf weight (*slw*, mg/cm2) collected from JHN WT and seven stomatal mutants grown under less restricted (LR) and more restricted water conditions (MR). All means are shown with standard deviations (±SD) and labelled with alphabetical combinations. The means shared similar alphabets were statistically non-significant (ns). Coefficient of variation, cv(a) and cv(b), denoted specifically for the main-plot treatment (water conditions) and the sub-plot treatment (mutants and the wild type), respectively. JHN WT (wild type) and four stomatal model mutants (SMM) are JHN826 (large-sized stomata), JHN3117 (small-sized stomata), JHN8756 (low-density stomata), JHN2447 (high-density stomata), and three drought-selected stomatal model mutants (DMM), JHN42, JHN352, JHN319.

	Lines	*sd* (mm^2)			*sz* (μm)			*si* (%)			*slw* (mg/cm^2)		
		LR	MR	Ave.Lines	LR	MR	Ave.Lines	LR	MR	Ave.Lines	LR	MR	Ave.Lines
	JHN (WT)	739 ± 78	731 ± 67	735 ± 69 b	13.5 ± 0.2	13.2 ± 0.1	13.4 ± 0.2 de	30.0 ± 2	33.4 ± 4	32 ± 3 b	5.5 ± 0.1	6.07 ± 1.1	5.8 ± 0.8
SMM	JHN3117 (SS)	787 ± 118	697 ± 79	746 ± 108 b	13.2 ± 0.4	13.3 ± 0.4	13.2 ± 0.4 e	31.5 ± 3	30.0 ± 2	31 ± 3 bc	5.6 ± 0.1	5.58 ± 0.4	5.6 ± 0.3
	JHN826 (LS)	728 ± 88	622 ± 145	679 ± 125 bc	14.5 ± 0.3	14.4 ± 0.4	14.4 ± 0.3 a	29.4 ± 3	28.2 ± 4	29 ± 3 cd	5.2 ± 0.2	5.77 ± 0.5	5.5 ± 0.5
	JHN8756 (LD)	672 ± 83	629 ± 69	651 ± 74 c	13.8 ± 0.4	14.0 ± 0.5	13.9 ± 0.4 bc	31.0 ± 3	29.7 ± 3	30 ± 3 bcd	5.6 ± 0.2	5.94 ± 0.4	5.8 ± 0.3
	JHN2447 (HD)	871 ± 51	846 ± 57	858 ± 52 a	13.7 ± 0.4	14.2 ± 0.3	14.0 ± 0.4 b	37.1 ± 2	34.8 ± 1	36 ± 2 a	5.4 ± 0.3	5.55 ± 0.0	5.5 ± 0.2
DMM	JHN42	667 ± 73	669 ± 26	668 ± 52 bc	13.5 ± 0.6	13.8 ± 0.4	13.6 ± 0.5 cd	28.3 ± 3	27.7 ± 3	28 ± 3 d	5.5 ± 0.4	5.66 ± 0.3	5.6 ± 0.3
	JHN319	706 ± 85	688 ± 51	697 ± 65 bc	13.5 ± 0.5	13.5 ± 0.4	13.5 ± 0.4 de	35.3 ± 5	35.3 ± 5	35 ± 5 a	5.6 ± 0.3	5.57 ± 0.2	5.6 ± 0.2
	JHN352	601 ± 134	635 ± 27	618 ± 92 c	13.4 ± 0.4	13.6 ± 0.2	13.5 ± 0.4 de	29.2 ± 2	28.8 ± 2	29 ± 2 cd	5.5 ± 0.2	5.52 ± 0.5	5.5 ± 0.4
	Ave. Water conditions	724 ± 111	688 ± 98		13.6 ± 0.5	13.8 ± 0.5		31.2 ± 4	30.8 ± 4		5.5 ± 0.3	5.71 ± 0.5	
	F-test												
	Water conditions		ns			ns			ns			ns	
	Lines		**			**			**			ns	
	Water conditions x Lir		ns			ns			ns			ns	
	cv(a)		13.0%			2.9%			7.2%			8.3%	
	cv(b)		12.7%			2.6%			10.0%			7.1%	
	Mean		706.3			13.7			31			5.6	

Supplementary Table 3: The effects of restricted water conditions on reduce plant height (cm) and reduce tillers/plant collected from JHN WT and seven stomatal mutants grown under less restricted (LR) and more restricted water conditions (MR). All means are shown with standard deviations (+SD) and labelled with alphabetical combinations. The means shared similar alphabets were statistically non-significant (ns). Coefficient of variation, cv(a) and cv(b), denoted specifically for the main-plot treatment (water conditions) and the sub-plot treatment (mutants and the wild type), respectively. JHN WT (wild type) and four stomatal model mutants (SMM) are JHN826 (large-sized stomata), JHN3117 (small-sized stomata), JHN8756 (low-density stomata), JHN2447 (high-density stomata), and three drought-selected stomatal model mutants (DMM), JHN42, JHN352, JHN319.

	Lines	Plant height (cm.)			%Reduce	Tillers/plant			%Reduce
		LR	MR	Ave.Lines	PH	LR	MR	Ave.Lines	Till
	JHN, WT	88.7 ± 4.1 a	71.5 ± 3.1 f	80.1 ± 9.6 a	19.4 ± 0.2 ab	14.0 ± 3.6	12.8 ± 2.3	13.4 ± 3.0 f	7.0 ± 7.9 c
SMM	JHN3117, SS	79.7 ± 2.8 cd	65.0 ± 3.7 g	72.3 ± 8.3 cd	18.5 ± 1.8 abc	15.7 ± 2.9	12.2 ± 1.8	13.9 ± 2.9 ef	22.0 ± 2.6 ab
	JHN826, LS	87.2 ± 2.8 ab	64.3 ± 7.0 g	75.8 ± 13.0 bc	26.3 ± 5.7 a	19.5 ± 5.0	13.7 ± 2.2	16.6 ± 4.8 de	28.6 ± 7.7 a
	JHN8756, LD	83.0 ± 2.4 bc	70.8 ± 5.3 f	76.9 ± 7.5 ab	14.7 ± 4.0 bc	21.5 ± 3.0	17.5 ± 3.8	19.5 ± 3.9 bc	19.2 ± 6.3 abc
	JHN2447, HD	77.0 ± 3.7 de	64.7 ± 5.4 g	70.8 ± 7.8 d	16.1 ± 2.9 bc	18.2 ± 2.9	15.7 ± 1.2	16.9 ± 2.5 cd	13.0 ± 7.1 bc
DMM	JHN42	80.2 ± 3.9 cd	72.0 ± 5.2 ef	76.1 ± 6.1 b	10.3 ± 2.1 c	24.3 ± 4.4	20.0 ± 2.8	22.2 ± 4.2 ab	17.4 ± 3.4 abc
	JHN319	80.0 ± 1.9 cd	71.0 ± 4.9 f	75.5 ± 5.9 bc	11.3 ± 4.0 bc	24.7 ± 5.0	22.2 ± 5.9	23.4 ± 5.4 a	10.9 ± 6.0 bc
	JHN352	79.0 ± 4.1 cd	69.2 ± 7.3 fg	74.1 ± 7.6 bcd	12.6 ± 4.8 bc	18.7 ± 3.9	17.3 ± 3.0	18.0 ± 3.4 cd	6.7 ± 3.3 c
	Ave. Water conditions	81.8 ± 4.9 A	68.6 ± 5.9 B			19.6 ± 5.1 A	16.4 ± 4.4 B		
	F-test								
	Water conditions		**				**		
	Lines		**		**		**		**
	Water conditions x Lines		**				ns		
	cv(a)		4.1%		22.2		10.7%		37.7
	cv(b)		6.0%				19.5%		
	Mean		75.2				18.0		

Note:

$$\%Reduce\_PH = \frac{PH(LR) - PH(MR)}{PH(LR)} x100$$

$$\%Reduce\_Till = \frac{Till(LR) - Till(MR)}{Till(LR)} x100$$

Supplementary Table 4: The effects of restricted water conditions on 50% days to flowering time (FD), % delayed flowering time (%Delay_FD), % filled grain (%FG), and % reduced filled grain (%Reduced_FG) collected from JHN WT and seven stomatal mutants grown under less restricted (LR) and more restricted water conditions (MR). All means are shown with standard deviations (±SD) and labelled with alphabetical combinations. The means shared similar alphabets were statistically non-significant (ns). Coefficient of variation, cv(a) and cv(b), denoted specifically for the main-plot treatment (water conditions) and the sub-plot treatment (mutants and the wild type), respectively. JHN WT (wild type) and four stomatal model mutants (SMM) are JHN826 (large-sized stomata), JHN3117 (small-sized stomata), JHN8756 (low-density stomata), JHN2447 (high-density stomata), and three drought-selected stomatal model mutants (DMM), JHN42, JHN352, JHN319.

		50 % time to Flowering			%Delay	% Filled grain			%Reduce
	Lines	LR	MR	Ave.Lines	FD	LR	MR	Ave.Lines	FG
	JHN (WT)	112 ± 2 abc	117 ± 4 a	114 ± 4 a	4.2 ± 1.0 bc	49.6 ± 6 abc	14.2 ± 2 ef	31.9 ± 19 ab	71.4 ± 0.8 c
	JHN3117 (SS)	100 ± 1 efg	107 ± 7 b-e	104 ± 6 b	6.9 ± 5.4 ab	44.8 ± 5 d	13.5 ± 2 efg	30.6 ± 16 bc	70.1 ± 2.0 cd
	JHN826 (LS)	98 ± 1 g	114 ± 8 ab	106 ± 10 b	15.9 ± 6.3 a	45.6 ± 5 d	10.5 ± 1 gh	28.0 ± 18 cd	77.1 ± 0.4 b
	JHN8756 (LD)	99 ± 0 g	105 ± 6 c-g	102 ± 5 b	6.4 ± 6.1 ab	47.5 ± 2 cd	11.0 ± 2 fgh	29.2 ± 19 cd	77.0 ± 3.9 b
SMM	JHN2447 (HD)	100 ± 1 fg	114 ± 4 ab	107 ± 8 b	14.0 ± 2.7 a	47.7 ± 7 bcd	7.5 ± 1 h	27.6 ± 21 d	84.4 ± 0.5 a
	JHN42	103 ± 4 d-g	110 ± 3 a-d	106 ± 5 b	6.9 ± 1.5 ab	51.3 ± 7 ab	13.4 ± 3 efg	32.4 ± 20 ab	74.1 ± 2.0 bc
	JHN319	100 ± 4 efg	105 ± 7 d-g	103 ± 6 b	4.2 ± 3.6 bc	48.0 ± 2 bcd	16.2 ± 2 e	30.7 ± 16 abc	66.3 ± 1.4 d
DMM	JHN352	106 ± 6 c-f	102 ± 5 efg	104 ± 5 b	-4.4 ± 0.8 c	52.2 ± 7 a	14.1 ± 2 efg	33.1 ± 20 a	73.1 ± 0.3 bc
	Ave. Water conditions	102 ± 5 B	109.1 ± 7 A			48.5 ± 6 A	12.7 ± 3 B		
	F-test								
	Water conditions		*				**		
	Lines		**		**		**		**
	Water conditions x Lines		*				**		
	cv(a)		5%		59.7		8.6%		2.4
	cv(b)		4%				14.2%		
	Mean		106				30.6		

$$\%Delay\_FD = \frac{DFL(LR) - DFL(MR)}{DFL(LR)} x100$$

$$\%Reduce\_FG = \frac{FG(LR) - FG(MR)}{FG(LR)} x100$$

Supplementary Table 5: The effects of restricted water conditions on water use efficiency (WUE), % Induced WUE (%Induce_WUE), shoot dried weight (SDW), and % reduced SDW (%Reduce_SDW) collected from JHN WT and seven stomatal mutants grown under less restricted (LR) and more restricted water conditions (MR). All means are shown with standard deviations (+SD) and labelled with alphabetical combinations. The means shared similar alphabets were statistically non-significant (ns). Coefficient of variation, cv(a) and cv(b), denoted specifically for the main-plot treatment (water conditions) and the sub-plot treatment (mutants and the wild type), respectively. JHN WT (wild type) and four stomatal model mutants (SMM) are JHN826 (large-sized stomata), JHN3117 (small-sized stomata), JHN8756 (low-density stomata), JHN2447 (high-density stomata), and three drought-selected stomatal model mutants (DMM), JHN42, JHN352, JHN319.

	Lines	WUE (mg/100ml) LR	WUE (mg/100ml) MR	WUE (mg/100ml) Ave.Lines	%Induce WUE	SDW(g/plant) LR	SDW(g/plant) MR	SDW(g/plant) Ave.Lines	%Reduce SDW
	JHN (WT)	61 ± 20 de	68 ± 5 de	65 ± 14 c	18 ± 31 abc	47.7 ± 15 abc	32.4 ± 2 def	40.0 ± 13 bc	28 ± 19 abc
SMM	JHN3117 (SS)	56 ± 8 e	63 ± 8 de	59 ± 8 c	12 ± 3 abc	43.7 ± 7 cd	29.7 ± 4 ef	36.7 ± 9 c	32 ± 2 abc
	JHN826 (LS)	70 ± 14 cde	56 ± 8 e	62 ± 12 c	-19 ± 5 c	54.7 ± 11 abc	26.7 ± 4 f	39.1 ± 16 c	51 ± 3 a
	JHN8756 (LD)	78 ± 13 cd	97 ± 7 ab	87 ± 15 a	27 ± 12 abc	60.6 ± 10 a	46.3 ± 4 bc	53.4 ± 10 a	23 ± 7 abc
	JHN2447 (HD)	69 ± 21 de	60 ± 4 de	64 ± 15 c	-7 ± 24 bc	53.6 ± 17 abc	28.6 ± 2 f	41.1 ± 17 bc	44 ± 15 ab
DMM	JHN42	76 ± 27 cd	114 ± 14 a	95 ± 28 a	59 ± 40 ab	59.5 ± 21 ab	54.0 ± 7 abc	56.7 ± 15 a	4 ± 24 bc
	JHN319	63 ± 19 de	104 ± 22 ab	84 ± 29 ab	68 ± 16 a	49.5 ± 15 abc	49.5 ± 11 abc	49.5 ± 12 ab	-2 ± 10 c
	JHN352	56 ± 22 e	89 ± 10 bc	73 ± 24 bc	76 ± 57 a	43.5 ± 18 cd	42.5 ± 5 cde	43.0 ± 12 bc	-7 ± 35 c
	Ave. Water conditions	66 ± 19 B	81 ± 23 A			51.5 ± 15 A	38.7 ± 11 B		
	F-test								
	Water conditions		**				**		
	Lines		**		**		**		**
	Water conditions x Lines		**				**		
	cv(a)		18		101		25.4		83
	cv(b)		21				23.8		
	Mean		74				45.0		

Note:

$$\%Induce\_WUE = \frac{WUE(MR) - WUE(LR)}{WUE(LR)} x100$$

$$\%Reduce\_SDW = \frac{SDW(LR) - SDW(MR)}{SDW(LR)} x100$$

Supplementary Table 6: Pearson correlation coefficient among stomatal mutants on % reduced plant height (%Reduce_PH), % reduced tiller/plant (%Reduce_Till),% delayed flowering (%Delay_FD), % reduced shoot dry weight (%Reduce_SDW), % induced water use efficiency (%Induce_WUE), and % reduced filled grains (%Reduce_FG) grown under less restricted (LR) and more restricted water conditions (MR). Correlation coefficients were tested for statistical significance at 0.05 (*) and 0.01 (**) levels.

Parameters	SD	%Reduce PH	%Reduce Till	%Delay FD	%Reduce SDW	%Induce WUE	%Reduce FG
SD	1.00						
%Reduce PH	0.21	1.00					
%Reduce Till	-0.05	0.56	1.00				
%Delay FD	0.56	0.59	0.70*	1.00			
%Reduce SDW	0.59	0.85**	0.59	0.84**	1.00		
%Induce WUE	-0.59	-0.85**	-0.59	-0.84**	-1.00**	1.00	
%Reduce FG	0.44	0.25	0.22	0.58	0.58	-0.58	1.00

Supplementary Table 7: The effects of restricted water conditions on maximum quantum yield of photosynthesis collected after 1, 7, 14, and 21 days after booting (DAB) from JHN WT and seven stomatal mutants grown under less restricted (LR) and more restricted water conditions (MR). All means are shown with standard deviations (+SD) and labelled with alphabetical combinations. The means shared similar alphabets were statistically non-significant (ns). Coefficient of variation, cv(a) and cv(b), denoted specific c.v. for the main-plot treatment (water conditions) and the sub-plot treatment (mutants and the wild type), respectively. JHN WT (wild type) and four stomatal model mutants (SMM) are JHN826 (large-sized stomata), JHN3117 (small-sized stomata), JHN8756 (low-density stomata), JHN2447 (high-density stomata), and three drought-selected stomatal model mutants (DMM), JHN42, JHN352, JHN319.

Lines		Maximum quantum yield of Photosystem II											
		1 DAB			7 DAB			14 DAB			21 DAB		
		LR	MR	Ave.Lines	LR	MR	Ave.Lines	LR	MR	Ave.Lines	LR	MR	Ave.Lines
	JHN, WT	0.78	0.78	0.78	0.82	0.79	0.80	0.79	0.76	0.78	0.82	0.78	0.80
SMM	JHN3117, SS	0.79	0.81	0.80	0.81	0.78	0.80	0.80	0.79	0.80	0.80	0.78	0.79
	JHN826, LS	0.80	0.80	0.80	0.82	0.80	0.81	0.79	0.80	0.79	0.79	0.79	0.79
	JHN8756, LD	0.80	0.79	0.79	0.80	0.79	0.80	0.80	0.79	0.80	0.78	0.78	0.78
	JHN2447, HD	0.78	0.80	0.79	0.80	0.77	0.79	0.79	0.79	0.79	0.78	0.79	0.78
DMM	JHN42	0.78	0.81	0.79	0.81	0.79	0.80	0.79	0.79	0.79	0.79	0.79	0.79
	JHN319	0.78	0.82	0.80	0.82	0.79	0.81	0.78	0.80	0.79	0.80	0.78	0.79
	JHN352	0.78	0.82	0.80	0.82	0.81	0.81	0.80	0.80	0.80	0.81	0.78	0.80
	Ave. Water conditions	0.79 B	0.80 A		0.81 A	0.79 B		0.79	0.79		0.80 A	0.78 B	
	F-test												
	Water conditions	*			*			ns			*		
	Lines	ns			ns			ns			ns		
	Water conditions x Lines	ns			ns			ns			ns		
	cv(a)	1.8%			2.9%			3.1%			1.2%		
	cv(b)	2.5%			2.4%			2.3%			2.0%		
	Mean	79%			80%			79%			79%		

Supplementary Table 8: The effects of restricted water conditions on SPAD index collected after 1, 7, 14, and 21 days after booting (DAB) from JHN WT and seven stomatal mutants grown under less restricted (LR) and more restricted water conditions (MR). All means are shown with standard deviations (+SD) and labelled with alphabetical combinations. The means shared similar alphabets were statistically non-significant (ns). Coefficient of variation, cv(a) and cv(b), denoted specifically for the main-plot treatment (water conditions) and the sub-plot treatment (mutants and the wild type), respectively. JHN WT (wild type) and four stomatal model mutants (SMM) are JHN826 (large-sized stomata), JHN3117 (small-sized stomata), JHN8756 (low-density stomata), JHN2447 (high-density stomata), and three drought-selected stomatal model mutants (DMM), JHN42, JHN352, JHN319.

	Lines	SPAD Index											
		1 DAB			7 DAB			14 DAB			21 DAB		
		LR	MR	Ave.Lines	LR	MR	Ave.Lines	LR	MR	Ave.Lines	LR	MR	Ave.Lines
	JHN (WT)	42.1	41.0	41.5 bc	46.9	47.1	47.0	46.5	48.9	47.7	45.3	50.2	47.8 bc
SMM	JHN3117 (SS)	40.8	41.1	40.9 bc	42.6	46.5	44.6	45.8	45.1	45.5	43.8	50.8	47.3 bc
	JHN826 (LS)	42.5	39.1	40.8 bc	48.2	45.0	46.6	49.3	46.1	47.7	46.5	47.9	47.2 bc
	JHN8756 (LD)	40.2	38.6	39.4 c	43.3	43.2	43.2	46.8	49.1	48.0	43.0	48.1	45.5 c
	JHN2447 (HD)	43.3	38.5	40.9 bc	45.8	48.6	47.2	46.0	47.7	46.8	46.1	47.0	46.5 c
DMM	JHN42	45.6	41.1	43.3 ab	45.2	42.3	43.7	46.0	50.2	48.1	48.8	50.1	49.4 ab
	JHN319	47.1	39.2	43.2 ab	45.6	45.6	45.6	50.0	47.4	48.7	50.6	48.4	49.5 ab
	JHN352	46.7	43.7	45.2 a	45.5	44.0	44.8	50.9	51.5	51.2	49.7	52.7	51.2 a
	Ave. Water conditions	43.5 A	40.3 B		45.4	45.3		47.7	48.2		46.7 B	49.4 A	
	F-test												
	Water conditions		**			ns			ns			**	
	Lines		**			ns			ns			**	
	Water conditions x Lines		ns			ns			ns			ns	
	cv(a)		4.3%			5.1%			3.1%			3.30%	
	cv(b)		6.5%			7.4%			7.0%			5.90%	
	Mean		42			45			48			48	

Supplementary Table 9: Stomata density counted and averaged (AVG) over combinations of individual field of view (FOV). For each stomatal mutant and JHN WT, the stomatal densities were averaged over the combinations of FOVs (*AVG*) and the six FOVs (*sd*, mm2). LR and MR signified the less and more restricted water conditions. JHN (wild type) and four stomatal model mutants (SMM) are JHN826 (large-sized stomata), JHN3117 (small-sized stomata), JHN8756 (low-density stomata), JHN2447 (high-density stomata), and three drought-selected stomatal model mutants (DMM), JHN42, JHN352, JHN319.

Water Conditions	Lines	Replication	FOV1	FOV2	FOV3	FOV4	FOV5	FOV6	Stomata AVG from ≤ 6 FOV	Stomata AVG / 6 FOV
LR	JHN	1	773	627	567				656	669
LR	JHN	2	858	717	669	828			768	768
LR	JHN	3	808	690	643	784	658		717	731
LR	JHN826	1	764	595	521				627	644
LR	JHN826	2	832	686	653	803			744	744
LR	JHN826	3	883	753	688	849	889	697	793	793
LR	JHN3117	1	748	580	526				618	644
LR	JHN3117	2	839	697	674	814			756	756
LR	JHN3117	3	731	605	586	706	562		638	657
LR	JHN8756	1	834	704					769	756
LR	JHN8756	2	822	672	647				714	731
LR	JHN8756	3	668	546	517	648			595	595
LR	JHN42	1	756	604					680	657
LR	JHN42	2	808	686	652				715	731
LR	JHN42	3	612	510	469	590			545	545
LR	JHN319	2	759	695	649				701	719
LR	JHN319	3	763	907	732	870			818	818
LR	JHN319	4	567	709	532	670	534		602	620
LR	JHN2447	1	874	1007					941	917
LR	JHN2447	2	846	997	827				890	905
LR	JHN2447	3	722	746	898	856			806	806
MR	JHN	1	676	566	532	655			607	607
MR	JHN	2	814	763	713				763	768
MR	JHN	3	790	662	626	748			707	706
MR	JHN	4	822	694	664	794	663	825	744	744
MR	JHN826	1	810	698	664				724	744
MR	JHN826	2	919	778					849	830
MR	JHN826	3	534	392	367	492			446	446
MR	JHN3117	1	768	669	614	774	817	596	706	706
MR	JHN3117	2	720	604	586				637	657
MR	JHN3117	3	916	778	745	882	910	751	830	830
MR	JHN8756	1	594	650	790	742			694	694
MR	JHN8756	2	752	635	614	725	692	671	682	682
MR	JHN8756	3	642	548					595	582
MR	JHN42	1	730	641	594				655	669
MR	JHN42	2	774	637	673	741	652	761	706	706
MR	JHN42	3	590	592	703	692	580	709	644	644
MR	JHN352	1	570	594	712				625	644
MR	JHN352	2	669	577	516	667			607	607
MR	JHN352	3	762	629					696	669
MR	JHN319	1	610	644	786				680	694
MR	JHN319	2	729	697	745	803			744	744
MR	JHN319	3	612	645	778	741			694	694
MR	JHN319	4	685	575	549	670	732		642	620
MR	JHN2447	1	981	850	824	964			905	905
MR	JHN2447	2	866	720	676				754	768
MR	JHN2447	3	924	804	795	897	792	919	855	855
MR	JHN2447	4	934	797	775				835	855
